# New Insight into the Prevalence and Risk Factors for Three Distinct Hoof Conformation Traits in UK Commercial Sheep Flocks

**DOI:** 10.3390/vetsci8090176

**Published:** 2021-08-30

**Authors:** Caroline M. Best, Janet Roden, Kate Phillips, Alison Z. Pyatt, Malgorzata C. Behnke

**Affiliations:** 1 Department of Veterinary Health & Animal Sciences, Harper Adams University, Newport, Shropshire TF10 8NB, UK; jroden@harper-adams.ac.uk (J.R.); kphillips@harper-adams.ac.uk (K.P.); mbehnke@harper-adams.ac.uk (M.C.B.); 2 International Office, Veterinary Medicines Directorate, Addlestone, Surrey KT15 3NB, UK; a.pyatt@vmd.gov.uk

**Keywords:** lameness, sheep, damaged, misshapen, overgrown, hoof conformation, prevalence, risk factors

## Abstract

Lameness in sheep continues to be a global health, welfare and economic concern. Damaged, misshapen or overgrown feet have the potential to cause lameness either directly, or indirectly. There is a lack of understanding of the predisposing factors for different hoof conformation traits in sheep. Our exploratory study aimed to investigate the prevalence of, and risk factors for, three distinct hoof conformation traits relating to the sole and heel, hoof wall, and hoof wall overgrowth. Feet of 400 ewes from four UK commercial sheep farms were inspected at four time points across 12 months. For each conformation trait, a four-point ordinal system was used to score each individual claw, and foot-level scores were calculated. We present 92.4% of foot-level observations to be affected by ≥1 conformation traits. Whilst hoof conformation traits were correlated to some degree, a unique set of sheep-, foot- and farm-level factors were associated with each distinct conformation trait. We provide, for the first time, key insight into the multifaceted and multifactorial aetiology of hoof conformation in sheep, building upon previous landmark studies. Our results inform hypotheses for future prospective studies investigating the risk factors for adverse hoof conformation in sheep.

## 1. Introduction

Lameness is one of the most significant health and welfare challenges facing sheep farmers worldwide. Sheep can become lame through infectious and non-infectious origins, but the majority of lameness in English sheep flocks is caused by footrot [1], an infectious bacterial disease caused by *Dichelobacter nodosus*. Footrot has two clinical presentations representing a continuum of infection: interdigital dermatitis (ID), which presents as inflammation of the interdigital skin, and severe footrot (SFR), which presents as the aggressive separation of hoof horn from the underlying sensitive tissue. Contagious ovine digital dermatitis (CODD) is also an infectious disease of significant concern, affecting approximately 50% of UK flocks [2,3], and was recently reported for the first time in Germany [4]. Non-infectious causes of lameness include white line disease (also known as shelly hoof), toe granulomas, foreign body penetration, soil balling, injuries to the limb, hoof cracks, and overgrown, damaged or misshapen claws. 

The ruminant hoof is a specialised epidermal structure, acting as an interface between the animal and its environment. The hoof is comprised of four key regions: periople, heel, sole horn, and wall horn [5]. The periople is a band of soft horn between the hoof wall and the coronary band, which extends plantar to form the heel; it is the rounded, soft and elastic area at the posterior part of the claw. Sole horn on the underside of the hoof is harder than the heel [6] and connects to the wall horn at the white line. Wall horn is the outer, dorsal surface of the hoof, which encapsulates the sensitive inner tissues and bones within the hoof. Wall horn is harder and less elastic than sole horn [7], and is produced through a complex process of epidermal cell differentiation and programmed cell death, or keratinisation and cornification. These processes act in synergy to harden horn tissue to provide mechanical strength essential for its role as the major weight bearing surface of the hoof. Wall horn grows continuously at approximately 3 mm per month [8], and its length is mediated by growth and wear; if growth is faster than wear, claws become overgrown [9]. Wall horn overgrowth is a common defect in sheep [10,11,12,13], typically presenting as an excess flap of horn part or fully covering the sole.

The significance of hoof conformation traits, or the shape, size and condition of hooves, has received little attention in sheep. It is known that sheep can display signs of impaired locomotion and stance due to poor hoof conformation, such as overgrown hooves [14,15], and damaged hoof horn [16]. Damaged and misshapen hooves are also more at risk of clinical footrot and resultant lameness [17]. Feet with misshapen and damaged sole and heel areas have elevated levels of *D. nodosus* present, with these sheep acting as subclinical carriers of infection [18]. Moreover, overgrown hoof horn appears to increase loads of *D. nodosus* found on infected feet [18], which could explain why hooves with wall overgrowth have an increased risk of clinical footrot [19]. 

Given the association between poor hoof conformation traits, bacterial load and lameness, understanding the risk factors for misshapen, damaged or overgrown hooves is integral to reducing lameness risk. Knowledge of risk factors could help predict changes to hoof integrity and higher risk of lameness, whilst also informing measures to promote good-structured and optimally-functioning hooves. To the authors’ knowledge, there have been no on-farm epidemiological studies explicitly investigating the risk factors for the prevalence nor severity of specific hoof conformation traits in commercial flocks. One 18-month study investigated the development of poor hoof conformation (or misshapen or damaged hooves) in sheep [17]. They speculated higher *D. nodosus* infection pressures at pasture to increase risk of poor conformation development. Conversely, they postulated lower ground and air temperatures, or dry underfoot conditions, to improve conformation. However, this landmark study did not differentiate between conformation of the sole and heel, and hoof wall, nor explicitly investigate a range of sheep-, and farm-level putative variables associated with distinct conformation traits. In cattle, studies investigating the environmental factors have made significant contributions to our understanding of the mechanical moderators of hoof conformation [6,20,21,22]. However, whilst these studies provide some indication of the likely agents involved in the manifestation of poor hoof conformation traits in sheep, extrapolation of knowledge between cattle and sheep is not always appropriate, especially considering their different management.

The aims of our study were to provide robust observational evidence of the prevalence of and potential risk factors for distinct hoof conformation traits in sheep from four commercial flocks over a 12-month period. To our knowledge, this is the first study of its kind.

## 2. Materials and Methods

### 2.1. Study Design and Data Collection

**Figure 1:**
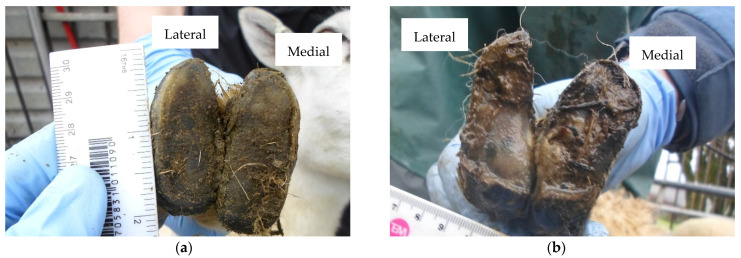
(**a**) Front foot with an undamaged sole and heel area and perfect shape (Foot-level score for sole and heel conformation = 0); (**b**) Front foot with a severely damaged sole and heel area of the medial claw (Foot-level score for sole and heel conformation = 3).

**Figure 2:**
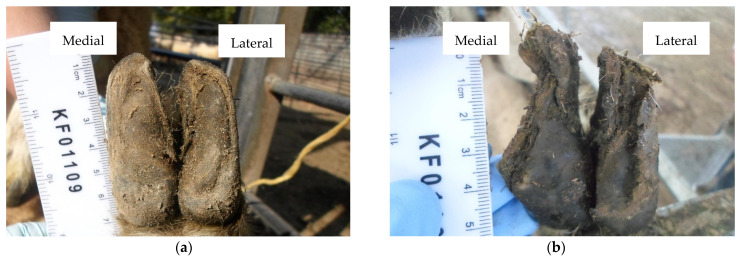
(**a**) Back foot with an undamaged hoof wall and perfect shape (Foot-level score for hoof wall conformation = 0); (**b**) Back foot with a severely misshapen and damaged hoof wall of the medial claw, and a moderately damaged lateral claw (Foot-level score for hoof wall conformation = 5).

**Figure 3:**
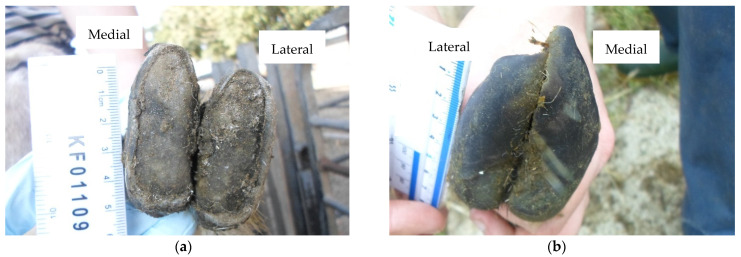
(**a**) Front foot with no hoof wall overgrowth present (Foot-level score for hoof wall overgrowth = 0); (**b**) Back foot with severely overgrown wall covering ≥ 75% of the sole of both claws (Foot-level score for hoof wall overgrowth = 6).

The study was a longitudinal, repeated cross-sectional field survey of four commercial sheep farms (identified as A–D) in England and Wales. Farm characteristics, study design and data collection are described in detail in [23]. Briefly, a minimum of 90 ewes were initially convenience selected from each flock, ensuring distribution between two age groups: <4 years and ≥4 years. All ewes were identified by ear tag numbers and marked for inclusion in the study. Farms were visited four times across a 12-month period: September 2019 (Visit 1), January 2020 (Visit 2), July 2020 (Visit 3) and September 2020 (Visit 4). Ewe age was recorded at the start of the study. At each visit, ewes were visually assessed for lameness [24] and body condition score (BCS) [25]. All four feet were first inspected for clinical disease, before all eight individual claws were assessed for three hoof conformation traits using four-point ordinal scoring systems (Table 1), as described in [18]: (1) sole and heel conformation, (2) hoof wall conformation and (3) hoof wall overgrowth. Examples of conformation scores are presented in Figure 1 (sole and heel), Figure 2 (hoof wall) and Figure 3 (hoof wall overgrowth). All examinations and assessments were made by a single observer (CMB) and recorded on paper recording sheets. 

Farm-level data were obtained from retrospective management practice questionnaires completed by each farmer. Data included farm soil type and flock footrot vaccination (Footvax®, MSD Animal Health, Merck & Co., Kenilworth, NJ, USA) status, in addition to estimates of pasture moisture, quality, type and sward height for each calendar month between August 2019 and September 2020. Meteorological data (average rainfall and temperature for each calendar month) were extracted from publicly available local UK MET Office station archives, as described in [23].

### 2.2. Data Preparation and Analysis

All data were manually entered into Microsoft Excel 2016 (Microsoft Corporation, Redmond, WA, USA). The final dataset comprised of 5672 foot-level observations and 1418 sheep-level observations obtained from 400 ewes across four farms. Total score at foot-level for each conformation trait was calculated by taking the sum of scores for the paired claws (lateral and medial) of that foot. The maximum score at foot-level per conformation trait was 6. Total score at sheep-level for each conformation trait was calculated by taking the sum of scores for all four feet. The maximum score at sheep-level per conformation trait was 24. Hoof conformation scores were treated as continuous data. The locomotion score was excluded from analyses due to the low prevalence of lameness in the ewes sampled. Due to low prevalence of SFR, presence of ID and SFR were combined into one category (‘Clinical disease’) for analysis. 

All analyses were performed in Genstat (VSN International, Hemel Hempstead, UK), and R statistical software package v 3.5.3 (R Foundation for Statistical Computing, Vienna, Austria). Associations between continuous and categorical variables were investigated using Kruskal–Wallis tests, and associations between categorical variables were investigated using Chi-squared tests. The relationship between hoof conformation scores were assessed by Spearman’s rank correlation coefficients (R_s_). Probability values of <0.05 were considered significant. 

### 2.3. Statistical Modelling

Univariable and multivariable linear mixed effects models were constructed to investigate the associations between sheep-, foot- and farm-level variables and each of the three hoof conformation traits at foot-level. The foot-level continuous outcome variables were: (1) sole and heel conformation, (2) hoof wall conformation and (3) hoof wall overgrowth, all coded 0–6. Models were constructed using the “lmer” function from the “lme4” package in R [26]. Models incorporated ‘Ewe’ and ‘Farm’ as random effects. The sheep-, foot- and farm-level variables considered as fixed-effects (explanatory variables) in the models are presented in Table 2. Due to only Farm B grazing ewes during Visit 2 (January 2020), pasture and meteorological data for all farms for the calendar month of Visit 2 were omitted from analyses. All candidate fixed-effects were first tested in univariable models, before constructing the multivariable model. Only variables with *p* < 0.2 at univariable level were selected to build the multivariable model. A manual backward elimination procedure was employed. Wald F and Wald-chi squared tests were used for significance testing, until only significant variables (*p* < 0.05) remained in the final model. Collinearity among fixed-effects was assessed using variance inflation factors (VIF) from the “car” package [27]. Collinear variables were not included in the same model. Instead, the most biologically plausible variable from the highly collinear pair was selected for inclusion into the model. The relative fit of models was assessed using Akaike Information Criterion (AIC). The model with the lowest AIC value was favoured. Residuals were inspected graphically for normality to ensure model assumptions were met. 

## 3. Results

### 3.1. Descriptive Results

#### 3.1.1. Prevalence and Severity of Hoof Conformation Traits at Sheep-Level

Ewes were persistently affected by ≥1 conformation trait; no ewes scored 0 for all three conformation traits throughout the duration of the study. Approximately 40% (*n* = 569/1418) of all sheep-level observations scored ≥ 1 for all three conformation traits concurrently. Almost 50% (*n* = 674/1418) of all sheep-level observations had ≥1 feet with misshapen and/or damaged sole and heel area. Over 95% (*n* = 1359/1418) had ≥1 feet with misshapen and/or damaged hoof wall, and over 89% (*n* = 1264/1418) had ≥1 feet with hoof wall overgrowth present. A summary of the scores for three hoof conformation traits at sheep-level are found in Table 3. 

#### 3.1.2. Prevalence and Severity of Hoof Conformation Traits at Foot-Level

Individual feet were persistently affected by ≥1 conformation trait (scores ≥ 1). Only 7.6% (*n* = 432/5672) of all foot-level observations scored 0 for all three conformation traits. Of those affected, approximately 13.4% (*n* = 700/5240) of foot-level observations scored ≥ 1 for all three conformation traits concurrently. Over 20% (*n* = 1182/5672) of foot-level observations had misshapen and/or damaged sole and heel areas. Almost three-quarters had misshapen and/or damaged hoof wall (73.6%, *n* = 4172/5672) or hoof wall overgrowth present (74.0%, *n* = 4196/5672). A summary of the scores for three hoof conformation traits at foot-level are found in Table 4. 

Front and back feet were disproportionately affected by hoof conformation traits (scores ≥ 1) (Table 5). Front feet were more often affected by hoof wall overgrowth (*p* < 0.001) and had higher mean wall overgrowth scores (*p* < 0.001). Back feet were more often affected by hoof wall conformation (*p* < 0.001) and had higher mean hoof wall scores (*p* < 0.001). No difference in sole and heel conformation prevalence was observed between front and back feet (*p* > 0.05), but back feet had higher mean sole and heel scores (*p* = 0.054), albeit a trend association.

**Figure 4:**
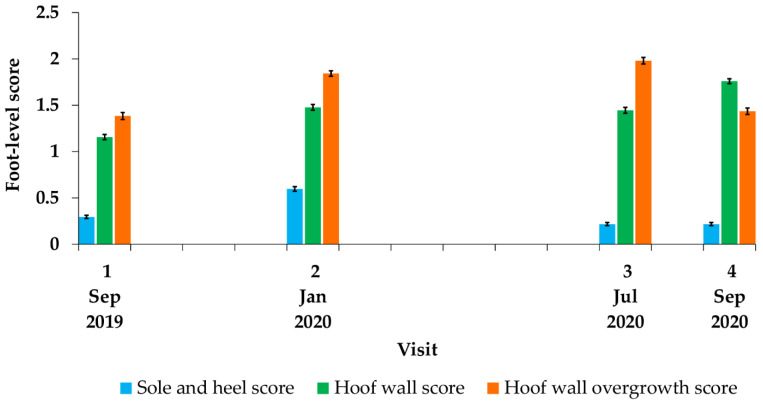
Mean foot-level conformation scores by visit for 5672 foot-level observations of 400 ewes. Visit 1: September 2019; Visit 2: January 2020; Visit 3; July 2020; Visit 4: September 2020. Error bars represent SEM.

Foot-level scores for conformation traits varied by farm (*p* < 0.001); higher sole and heel scores were observed on Farm A, higher hoof wall scores were observed on Farm C, and higher hoof wall overgrowth scores were observed on Farm B. Foot-level scores also changed over time (*p* < 0.001); highest scores for sole and heel conformation were observed at Visit 2, highest scores for hoof wall conformation were observed at Visit 4, and highest scores for hoof wall overgrowth were observed at Visit 3 (Figure 4).

#### 3.1.3. Associations between Hoof Conformation Traits at Foot-Level 

Sole and heel conformation and hoof wall conformation scores at foot-level showed a fair, positive correlation (R_s_ = 0.424, *p* < 0.001). Sole and heel conformation and hoof wall overgrowth scores showed a weak, positive association (R_s_ = 0.245, *p* = 0.001), similar to hoof wall conformation and hoof wall overgrowth scores (R_s_ = 0.157, *p* < 0.001).

### 3.2. Risk Factors Associated with Sole and Heel Conformation Score at Foot-Level

Univariable associations with sole and heel conformation score are presented in Supplementary Table S1. Six variables remained in the final multivariable model (Table 6). Back feet were more likely to have higher sole and heel scores than front feet (β = 0.06, 95% CI: 0.02–0.09). Furthermore, a foot was more likely to have higher sole and heel scores when ≥1 other feet of the ewe had scores ≥ 1. A foot was also more likely to have higher sole and heel scores when signs of footrot were present (β = 0.16, 95% CI: 0.08–0.23). Feet of sheep grazing pastures with a mixture of loamy and clay soils were more likely to have higher sole and heel scores, than those grazing on loamy soils (β = 0.23, 95% CI: 0.18–0.29). Additionally, feet of sheep grazing long pastures (approx. > 8 cm) one month previously were more likely to have higher sole and heel scores, than those grazing short sward heights (approx. 3 cm) (β = 0.17, 95% CI: 0.07–0.26). Feet of ewes were more likely to have higher scores at Visit 2 (January 2020), compared to Visit 1 (September 2019) (β = 0.23, 95% CI: 0.16–0.29). 

### 3.3. Risk Factors Associated with Hoof Wall Conformation Score at Foot-Level

Univariable associations with hoof wall conformation score are presented in Supplementary Table S2. Six variables remained in the final multivariable model (Table 7). Feet of ewes aged ≥ 4 years were more likely to have higher hoof wall scores than those aged < 4 years (β = 0.11, 95% CI: 0.02–0.20). Back feet were more likely to have higher hoof wall scores than front feet (β = 0.22, 95% CI: 0.16–0.27). Furthermore, a foot was more likely to have higher hoof wall scores when ≥ 1 other feet of the ewe had scores ≥ 1. Feet were also more likely to have higher hoof wall scores when ewes grazed damp (β = 0.36, 95% CI: 0.22–0.49), wet (β = 0.41, 95% CI: 0.18–0.64) or saturated (β = 0.64, 95% CI: 0.31–0.97) pastures one month previously, compared to dry pastures. In contrast, feet were more likely to have lower hoof wall scores when ewes grazed new grass leys alone (β = −0.23, 95% CI: −0.39–−0.08), or a mixture of permanent grassland and new leys (β = −0.81, 95% CI: −1.00–−0.63) one month previously, compared to permanent pasture only. Feet of ewes were more likely to have higher scores at Visit 3 (July 2020) (β = 0.35, 95% CI: 0.21–0.48) and Visit 4 (September 2020) (β = 0.58, 95% CI: 0.46–0.70), compared to Visit 1 (September 2019). 

### 3.4. Risk Factors Associated with Hoof Wall Overgrowth Score at Foot-Level

Univariable associations with hoof wall overgrowth score are presented in Supplementary Table S3. Ten variables remained in the final multivariable model (Table 8). Feet of ewes aged ≥ 4 years were more likely to have higher hoof wall overgrowth scores than those aged < 4 years (β = 0.12, 95% CI: 0.02–0.22). However, feet of ewes with BCS > 3.0 were more likely to have lower hoof wall overgrowth scores than those with BCS 3.0 (β = −0.10, 95% CI: −0.18–−0.03). Back feet were more likely to have lower hoof wall overgrowth scores than front feet (β = −0.57, 95% CI: −0.61–−0.52). A foot was more likely to have higher hoof wall overgrowth scores when ≥1 other feet of the ewe had scores ≥ 1. Feet were more likely to have lower hoof wall overgrowth scores when signs of footrot were present (β = −0.13, 95% CI: −0.23–−0.03). Feet of ewes were also more likely to have lower hoof wall overgrowth scores when grazing pastures with clay soils (β = −0.92, 95% CI: −1.27–−0.56), or a mixture of loamy and clay soils (β = −1.01, 95% CI: −1.43–−0.60), compared to those grazing loamy soils alone. Furthermore, feet were also more likely to have lower hoof wall overgrowth scores when ewes grazed damp (β = −0.38, 95% CI: −0.51–−0.25), or wet pastures (β = −1.29, 95% CI: −1.54–−1.05), one month previously, compared to dry pastures. Feet were more likely to have higher hoof wall overgrowth scores when ewes grazed a mixture of permanent grassland and new leys (β = 0.64, 95% CI: 0.48–0.79) one month previously, compared to permanent pasture only. Feet of sheep grazing long pastures (approx. >8 cm) were more likely to have lower hoof wall overgrowth scores than those grazing short sward heights (approx. 3 cm) (β = −0.55, 95% CI: −0.76–−0.35). Feet of ewes were more likely to have lower scores at Visit 3 (July 2020) (β = −0.50, 95% CI: −0.68–−0.33) and Visit 4 (September 2020) (β = −0.65, 95% CI: −0.78–−0.53), compared to Visit 1 (September 2019). 

A summary of the independent variables associated with increased or reduced foot-level scores for the three hoof conformation traits at multivariable level are presented in Table 9.

## 4. Discussion

We present novel results from the first observational study of three distinct hoof conformation traits in UK commercial sheep flocks. Our findings build upon a previous study [17] to detail the multifactorial aetiology of adverse hoof conformation in sheep and provide robust evidence for its multifaceted nature. Most notably, we highlight a lack of independence in hoof conformation traits at foot-level, and that different conformation traits, although correlated to some degree, have a unique combination of sheep-, foot- and farm-level risk factors at play.

We highlight three distinct adverse hoof conformation traits to be common in sheep. Whilst lameness in our sample of ewes was uncommon, we argue the chronicity of damaged and misshapen feet to be of significant health and welfare concern, particularly considering the potential for infectious disease and locomotion disorder. Additionally, chronically damaged or misshapen feet and the resultant irregular gait may cause long term irreversible damage to the bone structure of the foot and lower leg [28]. In line with current advice [29], we do not advocate the use of foot-trimming to improve hoof conformation in sheep, unlike in cattle. Trimming is counterproductive, not only increasing the risk of permanently misshapen or damaged hooves [30], but can accelerate the horn growth rate by approximately 4% [31]. It should be noted that no ewes from the flocks studied were foot trimmed. Instead, our study helps identify the factors affecting hoof conformation, thus highlighting potential managements informed by the understanding of risk. These are discussed in detail below.

Higher pasture moisture was a significant risk factor for hoof wall damage. Hoof horn of ewes grazing damp, wet or boggy pasture could have higher moisture content, reducing structural strength and increasing likelihood of maceration, wear and damage [32]. This is consistent with our finding that feet of ewes grazing damp or wet pasture were more likely to have lower wall overgrowth scores. Additionally, clay or mixed clay soils, and longer swards, which typically retain higher moisture levels, were associated with lower wall overgrowth scores, providing further supporting evidence to our hypothesis. However, this disagrees with previous work suggesting hoof horn length and wall overgrowth to increase with rainfall [9], and wet underfoot conditions [33].

In contrast, feet of ewes grazing loamy or mixed soils, or longer sward heights, were more likely to have higher sole and heel scores. This is concordant with our understanding that sole horn, which is softer than wall horn [7], can wear and become damaged through the abrasive action of well-drained, brashy calcareous soils and coarse, long, stalky grass. Longer sward heights have been associated with increased risk of white line disease [23], footrot [19], and general lameness in sheep [34]. Although sole and heel damage could be indicative of a previous episode of SFR, as discussed previously [18], our finding that feet with signs of footrot are more likely to have higher sole and heel conformation scores aligns with work highlighting higher loads of *D. nodosus* on feet with sole and heel damage present [18]. 

Feet of ewes grazing new leys, or a mixture of permanent and new leys, were more likely to have lower hoof wall scores, but higher wall overgrowth scores. This is contrary to previous findings highlighting these pastures to be associated with white line disease [23]. Without analysis of the micro- and macronutrients provided by different grazed pastures, the effects of nutrient supply are unclear. 

Ewes aged ≥ 4 years were more likely to have feet with higher hoof wall and hoof wall overgrowth scores. Hoof wall overgrowth has been observed more frequently in sheep aged > 4.5 years [35] and in ewes aged ≥ 2 years compared to yearlings and lambs [12], although the biological mechanisms are unclear. Greater hoof wall scores in older ewes could be the result of cumulative damage over time acting as precursors to hoof damage. Older ewes may be more prone to nutritional imbalances and laminitis, causing the production of low-quality hoof horn and greater susceptibility to damage. White line damage has been observed more frequently in ewes aged ≥ 4 years [23]. However, no association between age (≤4 years and >4 years) and degree of hoof shape or damage has previously been reported [17]. From our study, the awareness of increased risk of hoof damage and overgrowth in older ewes could inform managements to support hoof health in these ewes. Further work is required to understand the long-term effects of adverse hoof conformation traits on ewe lifetime performance. 

Some sheep in the study were never affected by poor conformation traits, albeit the minority. The genetic basis of individual variation in hoof conformation traits is poorly understood in sheep. Breed differences in hoof keratin structure and pigmentation may be linked with resistance to foot-related lameness [33]. As breed was confounded by farm in our study, breed differences in hoof conformation could not be explored. Further work identifying the heritability of hoof conformation traits is vital in understanding why some sheep may never become affected. Accordingly, this will help evaluate the importance of breeding replacements from good-footed ewes and how this can inform on-farm breeding policies. 

Interestingly, ewes with higher BCS (>3.0) were more likely to have feet with lower wall overgrowth scores, compared to fit ewes (BCS = 3.0). This is biologically plausible considering greater body weight, estimated to be around 12% of liveweight for every BCS score [36], exerted onto hoof horn could increase rate of wear. Ewes in poor condition (BCS 1–2.5) have been previously reported to have longer hoof horn than obese ewes (BCS 5) [9], but the different measurement system utilised invalidates comparison to our study.

For all hoof conformation traits, a foot was more likely to have higher scores when ≥1 other feet of the ewe had scores ≥ 1. This suggests that feet are not independent, and sheep- or farm-level factors are likely to play more important roles. Nonetheless, foot position was a significant risk factor for all three conformation traits, but varied between traits. Back feet were more likely to have higher scores for sole and heel, and hoof wall conformation. Back feet are typically exposed to higher levels of moisture and deeper faecal matter than front feet [37], and this can increase moisture content and elasticity as well as reduce structural strength and puncture resilience of hoof horn [6,7,21]. Although more body weight is loaded on front limbs than back limbs [38], hind limbs are often not squarely positioned; increasing hoof torsion, and coupled with the propulsive forces of the hind limb [39], could increase wear and damage to hoof integrity. This explanation is further supported by our finding that back feet were more likely to have lower scores for hoof wall overgrowth. This also aligns with previous studies observing a higher frequency of wall overgrowth in front feet of sheep [12,15], although contrary to findings from [13]. Therefore, asymmetry in hoof conformation scores observed between front and back feet are unlikely to be due to differences in growth rates, considering no differences have been observed previously in sheep [8,40]. 

Interestingly, feet with clinical disease were more likely to have lower scores for wall overgrowth. It is widely recognised that wall overgrowth proceeds a non-weight bearing event, such as footrot infection [9]. Considering hoof horn grows approximately 3 mm per month [8], a lag time between clinical disease and wall overgrowth would occur, but due to the nature of this study, this could not be explored. Furthermore, lameness was uncommon in our sample of ewes, where the majority of affected sheep were only showing mild signs of footrot, so ewes were weight-bearing on affected feet. Nonetheless, our findings corroborate those identifying the presence of wall overgrowth to be negatively associated with *D. nodosus* presence and load [18]. It is important to note that the farms recruited in this study had average flock lameness levels of ≤3%, and accordingly, had relatively low infection challenge. Further work is required to extend the study to flocks with higher lameness prevalence, to understand how risk factors for hoof conformation are influenced by disease challenge.

We report variation in the risk of hoof conformation traits over time. Feet of ewes at Visit 2 were more likely to have higher sole and heel scores than those at Visit 1. Three-quarters of the flocks studied were housed during Visit 2, which reflects the increase in risk during this time. Whilst warm, damp bedding is likely to cause the softening and maceration of sole and heel horn, other factors around parturition, such as hormone or nutrition, could play a role in reducing sole horn integrity [41]. Interestingly, feet were more likely to have higher hoof wall scores at Visits 3 and 4, but lower wall overgrowth scores during this time. We argue that lactation and nutrient partitioning could have considerable impact on hoof integrity, causing increased wear and subsequent damage to the hoof wall. Hoof wall conformation was the only trait in September 2020 to not return to levels similar to September 2019. This suggests that between-year variation in climatic conditions, and resultant pasture conditions, plays an important role for this trait. 

This study describes in detail the prevalence of, and risk factors for, three distinct hoof conformation traits at foot-level in commercial ewes from four UK sheep flocks, and as such, represents the largest such study of its kind. As with any longitudinal field survey, we cannot guarantee that the results are generalisable to all flocks. Due to the nature of the study, we cannot confirm the direction of causality of the associations between risk factors and hoof conformation trait score, but our findings provide reasonable insight into the likely candidates at play. Whilst the collection of pasture-based variables provided insightful data into specific conditions experienced by ewes, farmers’ estimations of variables may be impacted by recall bias, interpretation, and resultant observer bias. Future studies warrant the measurement of pasture conditions by a single observer at multiple time points across the month, in addition to objective measurements of soil moisture and temperature. Intensive observations of sheep at uniform sampling points could also prove valuable in further understanding the temporal dynamics of hoof conformation traits. Further measurements of hoof integrity, such as hoof hardness, could help elucidate the dynamics between hoof conformation, pasture moisture and the mechanical properties of hoof horn.

## 5. Conclusions

The aetiology of hoof conformation in sheep is complex. We have provided a detailed description of the hoof conformation traits affecting commercial ewes and have updated our understanding of the risk factors involved. We highlight a number of different sheep-, foot- and farm-level factors as key influences affecting the properties of the hoof. We propose wet underfoot conditions to be the most important environmental factor affecting hoof conformation in sheep, influencing the dynamics between moisture, abrasion, growth and wear. Further work is required to fully elucidate the causality of the risk factors identified, in order to inform early-intervention managements aimed at improving hoof conformation and increasing resilience to opportunistic infection and lameness. 

## Figures and Tables

**Table 1:** Four-point ordinal scoring systems for three hoof conformation traits: sole and heel conformation, hoof wall conformation and hoof wall overgrowth.

Trait	Description and Coding
Sole and heel	0 = Undamaged sole and heel area with a perfect shape
	1 = Mildly damaged and/or misshapen sole and heel area of the digit (<25%)
	2 = Moderately damaged and/or misshapen sole and heel area of the digit (≥25% to <75%)
	3 = Severely damaged and/or misshapen sole and heel area of the digit (≥75%)
Hoof wall	0 = Undamaged hoof wall with a perfect shape
	1 = Mildly damaged and/or misshapen hoof wall of the digit (<25%)
	2 = Moderately damaged and/or misshapen hoof wall of the digit (≥25% to <75%)
	3 = Severely damaged and/or misshapen hoof wall of the digit (≥75%)
Hoof wall overgrowth	0 = No hoof wall overgrowth
	1 = Mildly overgrown hoof wall covering the sole (<25%)
	2 = Moderately overgrown hoof wall covering the sole (≥25% to <75%)
	3 = Severely overgrown wall covering the sole (≥75%)

**Table 2:** Description of variables considered in analyses investigating associations with foot-level score for three conformation variables: sole and heel, hoof wall and hoof wall overgrowth.

Variable	Type	Description and Coding
*Sheep-level*		
Age	Categorical	Age of ewe at start of study 1 = <4 years 2 = ≥4 years
BCS	Categorical	Body condition score of ewe at time of visit 1 = 3.0 2 = <3.0 3 = >3.0
*Foot-level*		
Foot position	Categorical	1 = Front 2 = Back
Other feet affected by hoof conformation trait ^1^	Categorical	Number of other feet of ewe affected by hoof conformation trait 0 = No other feet with scores ≥ 1 1 = One other foot with score ≥ 1 2 = Two other feet with scores ≥ 1 3 = Three other feet with scores ≥ 1
Clinical disease	Categorical	Presence of footrot on the foot 0 = No footrot present 1 = ID and/or SFR present
*Farm-level*		
Vaccination status	Categorical	0 = Flock not vaccinated against footrot (Footvax^®^) 1 = Flock vaccinated against footrot (Footvax^®^)
Soil type	Categorical	1 = Loamy 2 = Clay 3 = Loamy/clay mix
Pasture moisture ^2^	Categorical	Average moisture of pasture grazed by ewes for calendar month 1 = Dry; hard ground, with little to no surface moisture 2 = Damp; firm ground, with moisture evident 3 = Wet; squelchy ground, but bears weight 4 = Saturated; boggy ground and bears no weight
Pasture quality ^2^	Categorical	Average quality of pasture grazed by ewes for calendar month 1 = Lush; approx. 80% rye grasses, mostly leaf 2 = Average; approx. 50% rye grasses, some stalk 3 = Poor; mostly stalk and weeds
Pasture type ^2^	Categorical	Average type of pasture grazed by ewes for calendar month 1 = Permanent grassland 2 = New grass ley 3 = Mix permanent and new ley
Sward height ^2^	Categorical	Average sward height of pasture grazed by ewes for calendar month 1 = Approx. 3 cm 2 = Approx. 8 cm 3 = Approx. > 8 cm
Rainfall ^2^	Continuous	Average rainfall (mm) for calendar month, extracted from local MET Office data
Temperature ^2^	Continuous	Average maximum temperature (°C) for calendar month, extracted from local MET Office data
*Time*		
Visit	Categorical	Sampling visit number 1 = September 2019 2 = January 2020 3 = July 2020 4 = September 2020

^1^ Hoof conformation trait as per the outcome variable (sole and heel, hoof wall or hoof wall overgrowth); ^2^ Two separate variables considered in analyses; variable for the calendar month of visit and variable lagged to the previous calendar month.

**Table 3:** Mean scores at sheep-level (*n* = 1418) for three hoof conformation traits from 400 ewes.

Trait	Mean Score All Observations (SD) ^1^	Sheep-Level Observations Score ≥ 1 *n*	Mean Score Affected Sheep Only (SD) ^2^	Max. Score ^3^
Sole and heel	1.36 (2.06)	674	2.87 (2.15)	14
Hoof wall	5.77 (3.22)	1359	6.02 (3.05)	17
Hoof wall overgrowth	6.65 (4.44)	1264	7.46 (4.01)	21

^1^ Mean score at sheep-level for all observations; ^2^ Mean score at sheep-level for observations with scores ≥ 1 only; ^3^ Maximum sheep-level score = 24; SD: standard deviation.

**Table 4:** Mean scores at foot-level (*n* = 5672) for three hoof conformation traits from 400 ewes.

Trait	Mean Score All Observations (SD) ^1^	Foot-Level Observations Score ≥ 1 *n*	Mean Score Affected Feet Only (SD) ^2^	Max. Score ^3^
Sole and heel	0.34 (0.78)	1182	1.64 (0.88)	6
Hoof wall	1.44 (1.14)	4172	1.96 (0.87)	6
Hoof wall overgrowth	1.66 (1.33)	4196	2.25 (1.04)	6

^1^ Mean score at foot-level for all observations; ^2^ Mean score at foot-level for observations with scores ≥ 1 only; ^3^ Maximum foot-level score = 6; SD: standard deviation.

**Table 5:** Mean scores at foot-level (*n* = 5672) by foot position for three hoof conformation traits from 400 ewes.

Trait	Foot-Level Observations Score ≥ 1 *n*			Mean Score (SD) ^2^		
	Front	Back	*p* Value ^1^	Front	Back	*p* Value ^3^
Sole and heel	566	616	0.102	0.31 (0.72)	0.37 (0.83)	0.054
Hoof wall	2025	2147	<0.001	1.34 (1.09)	1.54 (1.19)	<0.001
Hoof wall overgrowth	2269	1927	<0.001	1.93 (1.32)	1.40 (1.29)	<0.001

^1^ Chi-squared test *p* < 0.05 as significant; ^2^ Mean score at foot-level; ^3^ Kruskal–Wallis *p* < 0.05 as significant; SD: standard deviation.

**Table 6:** Final multivariable model of the associations with sole and heel conformation score for 5672 foot-level observations of 400 ewes.

Variable	*n*	%	β	Lower 95% CI	Upper 95% CI
Intercept			**0.13**	0.04	0.22
*Fixed effects*					
**Foot position**					
Front	2836	50.0	ref		
Back	2836	50.0	**0.06**	0.02	0.09
**Number of other feet with poor sole and heel conformation (scores ≥ 1)**					
No other feet affected	3306	58.3	ref		
One other foot affected	1418	25.0	**0.23**	0.19	0.28
Two other feet affected	716	12.6	**0.46**	0.40	0.52
Three other feet affected	232	4.1	**0.76**	0.66	0.86
**Clinical disease**					
No footrot present	5204	91.7	ref		
ID and/or SFR present	468	8.3	**0.16**	0.08	0.23
**Soil type**					
Loamy	1364	24.0	ref		
Clay	2856	50.4	0.03	−0.03	0.08
Loamy/clay mix	1452	25.6	**0.23**	0.18	0.29
**Sward height** **(lagged to previous calendar month)**					
Approx. 3 cm	2068	36.5	ref		
Approx. 8 cm	3208	56.6	−0.04	−0.10	0.03
Approx. >8 cm	396	7.0	**0.17**	0.07	0.26
**Visit**					
1 (Sep 2019)	1556	27.4	ref		
2 (Jan 2020)	1536	27.1	**0.23**	0.16	0.29
3 (Jul 2020)	1356	23.9	−0.01	−0.08	0.06
4 (Sep 2020)	1224	21.6	−0.01	−0.07	0.05
*Random terms*	*Variance*	*SD*			
**Ewe**	< 0.001	< 0.001			
**Farm**	< 0.001	< 0.001			

β: coefficient; CI: confidence interval for coefficient; bold coefficients are statistically significant at 0.05 as their CIs do not include 0; ref: baseline category for comparison.

**Table 7:** Final multivariable model of the associations with hoof wall conformation score for 5672 foot-level observations of 400 ewes.

Variable	*n*	%	β	Lower 95% CI	Upper 95% CI
Intercept			**0.79**	0.47	1.11
*Fixed effects*					
**Age**					
< 4 years	3528	62.2	ref		
≥ 4 years	2144	37.8	**0.11**	0.02	0.20
**Foot position**					
Front	2836	50.0	ref		
Back	2836	50.0	**0.22**	0.16	0.27
**Number of other feet with poor hoof wall conformation (scores ≥ 1)**					
No other feet affected	381	6.7	ref		
One other foot affected	903	15.9	**0.20**	0.07	0.33
Two other feet affected	1551	27.3	**0.45**	0.33	0.57
Three other feet affected	2837	50.0	**0.78**	0.66	0.91
**Pasture moisture (lagged to previous calendar month)**					
Dry (“hard”)	1604	28.3	ref		
Damp (“firm”)	2140	37.7	**0.36**	0.22	0.49
Wet (“squelchy”)	768	13.5	**0.41**	0.18	0.64
Saturated (“boggy”)	1160	20.5	**0.64**	0.31	0.97
**Pasture type** **(lagged to previous calendar month)**					
Permanent grassland	1768	31.2	ref		
New grass ley	3584	63.2	**−0.23**	−0.39	−0.08
Mix permanent and new ley	320	5.6	**−0.81**	−1.00	−0.63
**Visit**					
1 (Sep 2019)	1556	27.4	ref		
2 (Jan 2020)	1536	27.1	−0.18	−0.41	0.04
3 (Jul 2020)	1356	23.9	**0.35**	0.21	0.48
4 (Sep 2020)	1224	21.6	**0.58**	0.46	0.70
*Random terms*	*Variance*	*SD*			
**Ewe**	0.06	0.25			
**Farm**	0.07	0.26			

β: coefficient; CI: confidence interval for coefficient; bold coefficients are statistically significant at 0.05 as their CIs do not include 0; ref: baseline category for comparison.

**Table 8:** Final multivariable model of the associations with hoof wall overgrowth score for 5672 foot-level observations of 400 ewes.

Variable	*n*	%	β	Lower 95% CI	Upper 95% CI
Intercept			**2.53**	2.16	2.90
*Fixed effects*					
**Age**					
<4 years	3528	62.2	ref		
≥4 years	2144	37.8	**0.12**	0.02	0.22
**BCS**					
3.0	2816	49.6	ref		
<3.0	828	14.6	0.00	−0.10	0.10
>3.0	2028	35.8	**−0.10**	−0.18	−0.03
**Foot position**					
Front	2836	50.0	ref		
Back	2836	50.0	**−0.57**	−0.61	−0.52
**Number of other feet with hoof wall overgrowth present (scores ≥ 1)**					
No other feet affected	728	12.8	ref		
One other foot affected	672	11.8	**0.31**	0.19	0.43
Two other feet affected	900	15.9	**0.39**	0.27	0.52
Three other feet affected	3372	59.4	**1.06**	0.94	1.19
**Clinical disease**					
No footrot present	5204	91.7	ref		
ID and/or SFR present	468	8.3	**−0.13**	−0.23	−0.03
**Soil type**					
Loamy	1364	24.0	ref		
Clay	2856	50.4	**−0.92**	−1.27	−0.56
Loamy/clay mix	1452	25.6	**−1.01**	−1.43	−0.60
**Pasture moisture** **(lagged to previous calendar month)**					
Dry (“hard”)	1604	28.3	ref		
Damp (“firm”)	2140	37.7	**−0.38**	−0.51	−0.25
Wet (“squelchy”)	768	13.5	**−1.29**	−1.54	−1.05
Saturated (“boggy”)	1160	20.5	**−0.21**	−0.58	0.16
**Pasture type** **(lagged to previous calendar month)**					
Permanent grassland	1768	31.2	ref		
New grass ley	3584	63.2	0.09	−0.09	0.26
Mix permanent and new ley	320	5.6	**0.64**	0.48	0.79
**Sward height** **(lagged to previous calendar month)**					
Approx. 3 cm	2068	36.5	ref		
Approx. 8 cm	3208	56.6	0.00	−0.13	0.14
Approx. >8 cm	396	7.0	**−0.55**	−0.76	−0.35
**Visit**					
1 (Sep 2019)	1556	27.4	ref		
2 (Jan 2020)	1536	27.1	−0.09	−0.33	0.16
3 (Jul 2020)	1356	23.9	**−0.50**	−0.68	−0.33
4 (Sep 2020)	1224	21.6	**−0.65**	−0.78	−0.53
*Random terms*	*Variance*	*SD*			
**Ewe**	0.11	0.34			
**Farm**	0.02	0.14			

β: coefficient; CI: confidence interval for coefficient; bold coefficients are statistically significant at 0.05 as their CIs do not include 0; ref: baseline category for comparison.

**Table 9:** Summary of independent variables associated with increased or reduced foot-level scores for three conformation traits: sole and heel, hoof wall and hoof wall overgrowth.

Variable	Sole and Heel	>Hoof Wall	Hoof Wall Overgrowth
Age ≥ 4 years	NS	+	+
BCS < 3.0	NS	NS	NS
BCS > 3.0	NS	NS	−
Back foot position	+	+	−
Number of other feet of sheep affected by hoof conformation trait	+	+	+
ID and/or SFR present on foot	+	NS	−
Flock vaccinated against footrot (Footvax^®^)	NS	NS	NS
Clay soil type	NS	NS	−
Loamy/clay mix soil type	+	NS	−
Damp pasture	NS	+	−
Wet pasture	NS	+	−
Saturated pasture	NS	+	NS
Average pasture quality	NS	NS	NS
Poor pasture quality	NS	NS	NS
New grass ley	NS	−	NS
Mix permanent and new leys	NS	−	+
Approx. 8 cm sward height	NS	NS	NS
Approx. >8 cm sward height	+	NS	−
Rainfall	NS	NS	NS
Max temperature	NS	NS	NS
Visit 2 (January 2020)	+	NS	NS
Visit 3 (July 2020)	NS	+	−
Visit 4 (September 2020)	NS	+	−

NS: no significant association; + increased risk; − reduced risk.

## Data Availability

The data presented in this study are available on reasonable request from the corresponding author. The data is not publicly available as not all data from the study has been published yet.

## Supplementary Materials

The following are available online at https://www.mdpi.com/article/10.3390/vetsci8090176/s1, Table S1: Univariable analyses of the associations with sole and heel conformation score for 5672 foot-level observations of 400 ewes. Table S2: Univariable analyses of the associations with hoof wall conformation score for 5672 foot-level observations of 400 ewes. Table S3: Univariable analyses of the associations with hoof wall overgrowth score for 5672 foot-level observations of 400 ewes.
