# Supplementary material for: New Insight into the Prevalence and Risk Factors for Three Distinct Hoof Conformation Traits in UK Commercial Sheep Flocks
Source: Vet Sci. 2021 Aug 30;8(9):176. doi: 10.3390/vetsci8090176 (PMC8473389; doi:10.3390/vetsci8090176)
Supplement: Supplementary file 1 [file vetsci-08-00176-s001.zip › vetsci-1342054-supplementary.pdf]

Supplementary Materials

Table S1. Univariable analyses of the associations with sole and heel conformation score for 5672 foot-level observations of 400 ewes.

Variable	*n*	%	β	Lower 95% CI	Upper 95% CI
Sheep-level variables					
**Age**					
< 4 years	3528	62.2	ref		
≥ 4 years	2144	37.8	**0.08**	0.01	0.14
**BCS**					
3.0	2816	49.6	ref		
< 3.0	828	14.6	0.00	-0.06	0.07
> 3.0	2028	35.8	**0.10**	0.04	0.15
Foot-level variables					
**Foot position**					
Front	2836	50.0	ref		
Back	2836	50.0	**0.06**	0.02	0.10
**Number of other feet with poor sole and heel conformation (scores ≥ 1)**					
No other feet affected	3306	58.3	ref		
One other foot affected	1418	25.0	**0.29**	0.24	0.33
Two other feet affected	716	12.6	**0.56**	0.50	0.62
Three other feet affected	232	4.1	**0.91**	0.81	1.00
**Clinical disease**					
No FR disease present	5204	91.7	ref		
ID and/or SFR present	468	8.3	**0.28**	0.20	0.35
Farm-level variables					
**Vaccination status**					
Flock not vaccinated against footrot (Footvax®)	2816	49.6	ref		
Flock vaccinated against footrot (Footvax®)	2856	50.4	0.14	-0.21	0.48
**Soil type**					
Loamy	1364	24.0	ref		
Clay	2856	50.4	0.14	-0.04	0.33
Loamy/clay mix	1452	25.6	**0.38**	0.17	0.60
**Pasture moisture (calendar month of visit) (*n* = 4512)**					
Dry (“hard”)	996	22.1	ref		
Damp (“firm”)	2064	45.7	**0.07**	0.02	0.12
Wet (“squelchy”)	1452	32.2	**0.10**	0.04	0.16
Saturated (“boggy”)	0	0.0	-	-	-
**Pasture moisture (lagged to previous calendar month)**					
Dry (“hard”)	1604	28.3	ref		
Damp (“firm”)	2140	37.7	**0.11**	0.06	0.16

Wet (“squelchy”)	768	13.5	**0.20**	0.13	0.27
Saturated (“boggy”)	1160	20.5	**0.45**	0.40	0.51
**Pasture quality (calendar month of visit) (*n* = 4512)**					
Lush (~ 90% leafy rye grasses)	2812	62.3	ref		
Average (~ 50% rye grasses)	1700	37.7	**0.15**	0.09	0.21
Poor (mostly stalk and weeds)	0	0.0	-	-	-
**Pasture quality (lagged to previous calendar month)**					
Lush (~ 90% leafy rye grasses)	2516	44.4	ref		
Average (~ 50% rye grasses)	2760	48.7	**0.43**	0.34	0.51
Poor (mostly stalk and weeds)	396	7.0	**0.68**	0.56	0.80
**Pasture type (calendar month of visit) (*n* = 4512)**					
Permanent grassland	1008	22.3	ref		
New grass ley	752	16.7	0.13	-0.20	0.47
Mix permanent and new ley	2752	61.0	0.09	-0.24	0.42
**Pasture type (lagged to previous calendar month)**					
Permanent grassland	1768	31.2	ref		
New grass ley	3584	63.2	**-0.10**	-0.17	-0.02
Mix permanent and new ley	320	5.6	**-0.25**	-0.34	-0.16
**Sward height (calendar month of visit) (*n* = 4512)**					
Approx. 3 cm	1876	41.6	ref		
Approx. 8 cm	2324	51.5	-0.03	-0.07	0.02
Approx. > 8 cm	312	6.9	**-0.21**	-0.29	-0.12
**Sward height (lagged to previous calendar month)**					
Approx. 3 cm	2068	36.5	ref		
Approx. 8 cm	3208	56.6	**-0.12**	-0.17	-0.06
Approx. > 8 cm	396	7.0	**0.18**	0.09	0.27
**Rainfall (calendar month of visit)**	5672	100.0	0.00	0.00	0.00
**Rainfall (lagged to previous calendar month)**	5672	100.0	0.00	0.00	0.00
**Temperature (calendar month of visit)**	5672	100.0	**-0.03**	-0.03	-0.02
**Temperature (lagged to previous calendar month)**	5672	100.0	**-0.03**	-0.03	-0.02
Time variable					
**Visit**					
1 (Sep 2019)	1556	27.4	ref		
2 (Jan 2020)	1536	27.1	**0.30**	0.25	0.35
3 (Jul 2020)	1356	23.9	**-0.09**	-0.14	-0.03
4 (Sep 2020)	1224	21.6	**-0.08**	-0.13	-0.02

β: estimate; CI: confidence interval for estimate; bold estimates are statistically significant at 0.05 as their CIs do not include 0; ref: baseline category for comparison.

Table S2. Univariable analyses of the associations with hoof wall conformation score for 5672 foot-level observations of 400 ewes.

**Variable**	***n***	**%**	**β**	**Lower 95% CI**	**Upper 95% CI**
Sheep-level variables					
**Age**					
< 4 years	3528	62.2	ref		
≥ 4 years	2144	37.8	**0.13**	0.02	0.24
**BCS**					
3.0	2816	49.6	ref		
< 3.0	828	14.6	**0.15**	0.05	0.25
> 3.0	2028	35.8	0.03	-0.05	0.11
Foot-level variables					
**Foot position**					
Front	2836	50.0	ref		
Back	2836	50.0	**0.20**	0.15	0.26
**Number of other feet with poor hoof wall conformation (scores ≥ 1)**					
No other feet affected	381	6.7	ref		
One other foot affected	903	15.9	**0.28**	0.15	0.41
Two other feet affected	1551	27.3	**0.63**	0.50	0.75
Three other feet affected	2837	50.0	**1.11**	0.99	1.22
**Clinical disease**					
No FR disease present	5204	91.7	ref		
ID and/or SFR present	468	8.3	**0.28**	0.17	0.39
Farm-level variables					
**Vaccination status**					
Flock not vaccinated against footrot (Footvax®)	2816	49.6	ref		
Flock vaccinated against footrot (Footvax®)	2856	50.4	-0.12	-0.55	0.32
**Soil type**					
Loamy	1364	24.0	ref		
Clay	2856	50.4	-0.07	-0.35	0.22
Loamy/clay mix	1452	25.6	**-0.40**	-0.73	-0.07
**Pasture moisture (calendar month of visit) (*n* = 4512)**					
Dry ("hard")	996	22.1	ref		
Damp ("firm")	2064	45.7	**0.09**	0.01	0.17
Wet ("squelchy")	1452	32.2	**-0.31**	-0.41	-0.21
Saturated ("boggy")	0	0.0	-	-	-
**Pasture moisture (lagged to previous calendar month)**					
Dry ("hard")	1604	28.3	ref		
Damp ("firm")	2140	37.7	**-0.13**	-0.21	-0.05
Wet ("squelchy")	768	13.5	**-0.17**	-0.28	-0.07
Saturated ("boggy")	1160	20.5	**-0.13**	-0.21	-0.04
**Pasture quality**					

**(calendar month of visit) (*n* = 4512)**					
Lush (~ 90% leafy rye grasses)	2812	62.3	ref		
Average (~ 50% rye grasses)	1700	37.7	**0.44**	0.36	0.53
Poor (mostly stalk and weeds)	0	0.0	-	-	-
**Pasture quality (lagged to previous calendar month)**					
Lush (~ 90% leafy rye grasses)	2516	44.4	ref		
Average (~ 50% rye grasses)	2760	48.7	**-0.17**	-0.29	-0.05
Poor (mostly stalk and weeds)	396	7.0	**-0.19**	-0.36	-0.01
**Pasture type (calendar month of visit) (*n* = 4512)**					
Permanent grassland	1008	22.3	ref		
New grass ley	752	16.7	**-0.74**	-0.83	-0.66
Mix permanent and new ley	2752	61.0	**-0.08**	-0.15	-0.02
**Pasture type (lagged to previous calendar month)**					
Permanent grassland	1768	31.2	ref		
New grass ley	3584	63.2	**-0.50**	-0.60	-0.39
Mix permanent and new ley	320	5.6	**-0.62**	-0.75	-0.49
**Sward height (calendar month of visit) (*n* = 4512)**					
Approx. 3 cm	1876	41.6	ref		
Approx. 8 cm	2324	51.5	**0.61**	0.54	0.67
Approx. > 8 cm	312	6.9	**0.53**	0.39	0.67
**Sward height (lagged to previous calendar month)**					
Approx. 3 cm	2068	36.5	ref		
Approx. 8 cm	3208	56.6	-0.08	-0.16	0.00
Approx. > 8 cm	396	7.0	**-0.20**	-0.32	-0.07
**Rainfall (calendar month of visit)**	5672	100.0	**-0.01**	-0.01	-0.01
**Rainfall (lagged to previous calendar month)**	5672	100.0	**0.01**	0.01	0.01
**Temperature (calendar month of visit)**	5672	100.0	**-0.03**	-0.04	-0.02
**Temperature (lagged to previous calendar month)**	5672	100.0	-0.01	-0.01	0.00
Time variable					
**Visit**					
1 (Sep 2019)	1556	27.4	ref		
2 (Jan 2020)	1536	27.1	**0.32**	0.25	0.40
3 (Jul 2020)	1356	23.9	**0.30**	0.22	0.37
4 (Sep 2020)	1224	21.6	**0.61**	0.53	0.69

β: estimate; CI: confidence interval for estimate; bold estimates are statistically significant at 0.05 as their CIs do not include 0; ref: baseline category for comparison.

Table S3. Univariable analyses of the associations with hoof wall overgrowth score for 5672 foot-level observations of 400 ewes.

Variable	*n*	%	β	Lower 95% CI	Upper 95% CI
Sheep-level variables					
**Age**					
< 4 years	3528	62.2	ref		
≥ 4 years	2144	37.8	**0.17**	0.05	0.28
**BCS**					
3.0	2816	49.6	ref		
< 3.0	828	14.6	**0.51**	0.41	0.62
> 3.0	2028	35.8	**-0.46**	-0.54	-0.37
Foot-level variables					
**Foot position**					
Front	2836	50.0	ref		
Back	2836	50.0	**-0.54**	-0.60	-0.49
**Number of other feet with hoof wall overgrowth present (scores ≥ 1)**					
No other feet affected	728	12.8	ref		
One other foot affected	672	11.8	**0.57**	0.45	0.68
Two other feet affected	900	15.9	**0.73**	0.62	0.85
Three other feet affected	3372	59.4	**1.72**	1.63	1.82
**Clinical disease**					
No FR disease present	5204	91.7	ref		
ID and/or SFR present	468	8.3	**-0.38**	-0.50	-0.26
Farm-level variables					
**Vaccination status**					
Flock not vaccinated against footrot (Footvax®)	2816	49.6	ref		
Flock vaccinated against footrot (Footvax®)	2856	50.4	-0.68	-1.95	0.60
**Soil type**					
Loamy	1364	24.0	ref		
Clay	2856	50.4	**-0.82**	-1.23	-0.41
Loamy/clay mix	1452	25.6	**-1.59**	-2.07	-1.12
**Pasture moisture (calendar month of visit) (*n* = 4512)**					
Dry ("hard")	996	22.1	ref		
Damp ("firm")	2064	45.7	**-0.62**	-0.69	-0.54
Wet ("squelchy")	1452	32.2	**-1.08**	-1.18	-0.98
Saturated ("boggy")	0	0.0	-	-	-
**Pasture moisture (lagged to previous calendar month)**					
Dry ("hard")	1604	28.3	ref		
Damp ("firm")	2140	37.7	**-0.57**	-0.65	-0.50
Wet ("squelchy")	768	13.5	**-1.50**	-1.60	-1.40
Saturated ("boggy")	1160	20.5	**0.24**	0.15	0.32
**Pasture quality**					

**(calendar month of visit) (*n* = 4512)**					
Lush (~ 90% leafy rye grasses)	2812	62.3	ref		
Average (~ 50% rye grasses)	1700	37.7	**-0.13**	-0.22	-0.04
Poor (mostly stalk and weeds)	0	0.0	-	-	-
**Pasture quality (lagged to previous calendar month)**					
Lush (~ 90% leafy rye grasses)	2516	44.4	ref		
Average (~ 50% rye grasses)	2760	48.7	**1.93**	1.81	2.05
Poor (mostly stalk and weeds)	396	7.0	**1.13**	0.96	1.30
**Pasture type (calendar month of visit) (*n* = 4512)**					
Permanent grassland	1008	22.3	ref		
New grass ley	752	16.7	-1.12	-3.94	1.69
Mix permanent and new ley	2752	61.0	-0.53	-3.34	2.29
**Pasture type (lagged to previous calendar month)**					
Permanent grassland	1768	31.2	ref		
New grass ley	3584	63.2	**0.81**	0.67	0.95
Mix permanent and new ley	320	5.6	**0.58**	0.46	0.69
**Sward height (calendar month of visit) (*n* = 4512)**					
Approx. 3 cm	1876	41.6	ref		
Approx. 8 cm	2324	51.5	**0.14**	0.07	0.20
Approx. > 8 cm	312	6.9	**-0.25**	-0.39	-0.11
**Sward height (lagged to previous calendar month)**					
Approx. 3 cm	2068	36.5	ref		
Approx. 8 cm	3208	56.6	**-0.41**	-0.49	-0.33
Approx. > 8 cm	396	7.0	**-1.10**	-1.22	-0.97
**Rainfall (calendar month of visit)**	5672	100.0	0.00	0.00	0.00
**Rainfall (lagged to previous calendar month)**	5672	100.0	0.00	0.00	0.00
**Temperature (calendar month of visit)**	5672	100.0	**0.12**	0.11	0.13
**Temperature (lagged to previous calendar month)**	5672	100.0	**-0.02**	-0.02	-0.01
Time variable					
**Visit**					
1 (Sep 2019)	1556	27.4	ref		
2 (Jan 2020)	1536	27.1	**0.46**	0.38	0.53
3 (Jul 2020)	1356	23.9	**0.62**	0.53	0.70
4 (Sep 2020)	1224	21.6	0.05	-0.04	0.13

β: estimate; CI: confidence interval for estimate; bold estimates are statistically significant at 0.05 as their CIs do not include 0; ref: baseline category for comparison.
